# The value of the current histological scores and classifications of ANCA glomerulonephritis in predicting long-term outcome

**DOI:** 10.1093/ckj/sfae125

**Published:** 2024-05-06

**Authors:** Matteo Stella, Laura Locatelli, Filippo Maria Sala, Francesco Reggiani, Marta Calatroni, Vincenzo L'Imperio, Fabio Pagni, Umberto Maggiore, Gabriella Moroni, Renato Alberto Sinico

**Affiliations:** Department of Biomedical Sciences, Humanitas University, Pieve Emanuele, Milan, Italy; IRCCS Humanitas Research Hospital Rozzano, Milan, Italy; IRCCS Humanitas Research Hospital Rozzano, Milan, Italy; Nephrology Unit, ASST della Brianza, Vimercate Hospital, Vimercate, Italy; Department of Biomedical Sciences, Humanitas University, Pieve Emanuele, Milan, Italy; IRCCS Humanitas Research Hospital Rozzano, Milan, Italy; Department of Biomedical Sciences, Humanitas University, Pieve Emanuele, Milan, Italy; IRCCS Humanitas Research Hospital Rozzano, Milan, Italy; Department of Medicine and Surgery, Pathology, University of Milano-Bicocca, Fondazione IRCCS San Gerardo dei Tintori, Monza, Italy; Department of Medicine and Surgery, Pathology, University of Milano-Bicocca, Fondazione IRCCS San Gerardo dei Tintori, Monza, Italy; Department of Medicine and Surgery, Nephrology Unit, Azienda Ospedaliera-Universitaria Parma, University of Parma, Parma, Italy; Department of Biomedical Sciences, Humanitas University, Pieve Emanuele, Milan, Italy; IRCCS Humanitas Research Hospital Rozzano, Milan, Italy; IRCCS Humanitas Research Hospital Rozzano, Milan, Italy

**Keywords:** ANCA, Berden, glomerulonephritis, Mayo Clinic Chronicity Score, Renal Risk Score

## Abstract

**Background:**

Three different histological scores—histopathologic classification (Berden), Renal Risk Score (RRS) and the Mayo Clinic Chronicity Score (MCCS)—for anti-neutrophil cytoplasmic antibody (ANCA)-associated glomerulonephritis (ANCA-GN) were compared to evaluate their association with patient and kidney prognosis of ANCA-GN.

**Methods:**

Patients aged >18 years with at least 1 year of follow-up and biopsy-proven ANCA-GN entered this retrospective study. Renal biopsies were classified according to Berden's classification, RRS and MCCS. The first endpoint was end-stage kidney disease (ESKD), defined as chronic dialysis or estimated glomerular filtration rate <15 mL/min/1.73 m^2^. The second endpoint was ESKD or death.

**Results:**

Of 152 patients 84 were males, with median age of 63.8 years and followed for 46.9 (interquartile range 12.8–119) months, 59 (38.8%) reached the first endpoint and 20 died. The Kaplan–Meier curves showed that Berden and RRS were associated with first (Berden: *P* = .004, RRS: *P* < .001) and second (Berden: *P* = .001, RRS: *P* < .001) endpoint, MCCS with the first endpoint only when minimal + mild vs moderate + severe groups were compared (*P* = .017), and with the second endpoint (*P* < .001). Among the clinical/histological presentation features, arterial hypertension [odds ratio (OR) = 2.75, confidence interval (95% CI) 1.50–5.06; *P* = .0011], serum creatinine (OR = 1.17, 95% CI 1.09–1.25; *P* < .0001), and the percentage of normal glomeruli (OR = 0.97, 95% CI 0.96–0.99; *P* = .009) were the independent predictors of ESKD at multivariate analysis. When the three scores were included in multivariate analysis, RRS (OR = 2.21, 95% CI 1.15–4.24; *P* = .017) and MCCS (OR = 2.03, 95% CI 1.04–3.95; *P* = .037) remained predictive of ESKD, but Berden (OR = 1.17, 95% CI 0.62–2.22; *P* = .691) did not.

**Conclusion:**

RRS and MCCS scores were independent predictors of kidney survival together with high serum creatinine and arterial hypertension at diagnosis, while Berden classification was not.

## Graphical Abstract

**Graphical Abstract:**
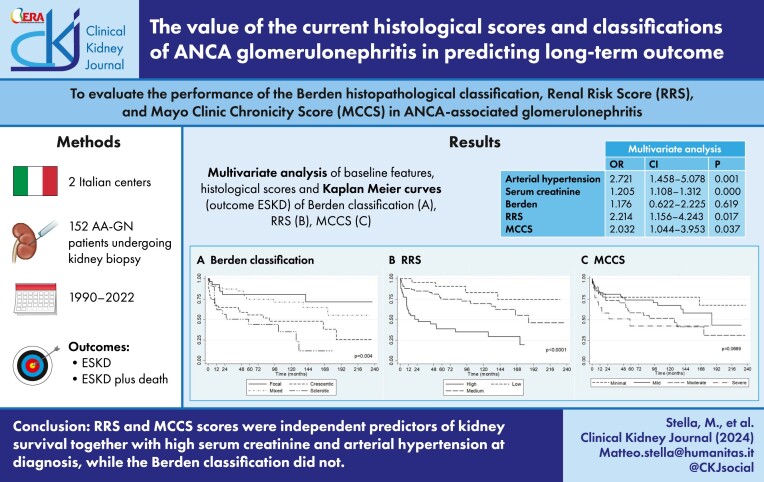

KEY LEARNING POINTS **What was known:**

- Anti-neutrophil cytoplasmic antibody (ANCA)-associated vasculitides (AAV) are a group of rare systemic autoimmune diseases that can often affect the kidneys, frequently presenting with rapidly progressive renal insufficiency that requires prompt and aggressive treatment to avoid irreversible renal dysfunction.
- Different histological scoring systems have been proposed to evaluate their association with ANCA-associated glomerulonephritis (ANCA-GN) outcome.

**This study adds:**

- Serum creatinine and arterial hypertension at diagnosis together with Renal Risk Score (RRS) and the Mayo Clinic Chronicity Score (MCCS) predicted kidney survival at multivariate analysis, while Berden did not.

**Potential impact:**

- The more recent proposed scores (RRS and MCCS), including not only glomerular lesions but also interstitial and tubular abnormalities, allow a better stratification of patients with ANCA-GN.
- The RRS seems to be more accurate, probably because it includes the kidney function.

## INTRODUCTION

Anti-neutrophil cytoplasmic antibody (ANCA)-associated vasculitides (AAV) are a group of rare systemic autoimmune diseases that can often affect the kidneys causing necrotizing inflammation of small blood vessels, endothelial injury and tissue

damage [1]. A typical and diagnostic finding is the positivity of ANCA against either proteinase 3 (PR3-ANCA) or myeloperoxidase (MPO-ANCA) [2].

AAV are clinically classified in three main forms: granulomatosis with polyangiitis (GPA), microscopic polyangiitis (MPA) and eosinophilic granulomatosis with polyangiitis (EGPA). Except for the EGPA, in which kidney involvement is infrequent (20%–30% of the cases) [1, 3, 4], in the other forms renal involvement occurs in 50%–90% of cases [1, 5, 6], characterized by microscopic hematuria, various degree of proteinuria and a substantial decrease of the estimated glomerular filtration rate (eGFR), typically in the form of a rapidly progressive glomerulonephritis (RPGN) [7]. If not diagnosed in a timely manner and treated rapidly, end-stage kidney disease (ESKD) occurs and requires renal replacement therapy. In these cases, death is not infrequent [8, 9].

The kidney biopsy is a milestone in the diagnosis of ANCA-AAV; histological findings on renal biopsy have an important prognostic value. In particular, the grade of chronic damage in the kidney biopsy at presentation is considered one of the principal predictors of the renal prognosis and of overall patient survival [10].

To categorize the histology of ANCA-GN and determine which of the histological lesions are more predictive of renal outcome, the European Vasculitis Study Group (EUVAS) in 2010 proposed a classification based on four histological classes based on glomerular lesions only: focal, crescentic, mixed and sclerotic classes [11]. They demonstrated that the renal prognosis at 1 and at 5 years progressively worsened from focal to sclerotic classes. In the last years, however, the prognostic value of this classification has been questioned [12, 13].

Some other groups have subsequently proposed new histological scores to predict the renal outcome of ANCA-GN.

The Mayo Clinic Chronicity Score (MCCS) proposed by Sethi *et al*. [14], to classify all types of glomerular diseases, considers the degree of global and segmental glomerulosclerosis, tubular atrophy, interstitial fibrosis and arteriosclerosis/arteriolosclerosis. The Renal Risk Score (RRS) conceived by Brix *et al*. [15] evaluates as parameters the percentage of normal glomeruli, the grade of tubular atrophy and interstitial fibrosis, and the estimated GFR (eGFR) at the time of biopsy.

The availability of a simple tool to accurately predict prognosis in ANCA-GN might enable a more tailored therapeutic approach.

In this paper, we aimed to evaluate the clinical histological characteristics, treatment and patient/kidney prognosis in a large cohort with biopsy-proven ANCA-associated glomerulonephritis (ANCA-GN). In particular, the objectives of the study were: (i) to assess the risk of ESKD and that of ESKD or death; (ii) to establish the clinical/histological predictors of ESKD at univariate and at multivariate analysis among the clinical histological features at presentation of ANCA-GN; and (iii) to compare the power of the three different histological above-mentioned scores [11, 14, 15] in predicting ESKD and death of ANCA-GN patients.

## MATERIALS AND METHODS

### Study population

We selected for this study all patients who developed renal involvement during AAV and underwent a kidney biopsy receiving a diagnosis of ANCA-GN in two Nephrological Italian centers (IRCCS Fondazione San Gerardo dei Tintori, Monza and Humanitas Research Hospital, Milan) from 1990 to 2022. Inclusion criteria were: (i) age >18 years, (ii) kidney biopsy with diagnosis of ANCA-GN and a minimum of 10 glomeruli in the kidney specimen and (iii) a follow-up of at least 1 year. Patients with secondary forms of ANCA-GN were excluded.

### Data collection

For each patient, we collected demographic data (age at diagnosis of ANCA-GN, sex, blood pressure), clinical renal and extra renal vasculitic signs and symptoms at diagnosis, throughout the follow-up and at last observation. In particular, the Birmingham Vasculitis Activity Score (BVAS) was employed to assess the activity of the vasculitis [16] and the following laboratory tests were recorded: serum creatinine (mg/dL), 24 h proteinuria (g/day), the eGFR [using Chronic Kidney Disease Epidemiology Collaboration (CKD-EPI) formula, mL/min/1.73 m^2^], microscopic hematuria at urinary sediment, ANCA type and hemoglobin (g/dL).

The therapy administered at diagnosis included corticosteroids therapy (methylprednisolone pulses or oral prednisone) and immunosuppressive agents (cyclophosphamide, azathioprine and mycophenolate, rituximab, methotrexate); some of these immunosuppressive agents were employed also as maintenance therapy.

For kidney biopsy, we collected the total number of glomeruli, the percentage of normal glomeruli, of those globally and segmentally sclerotic, of those with crescents and those with fibrinoid necrosis. In the vascular compartment we assessed the presence of endarteritis and in the tubulo-interstitial area the amount of tubular atrophy and interstitial fibrosis.

Renal biopsies were classified according to Berden *et al*. [11] into four classes:

- Sclerotic class: >50% globally sclerotic glomeruli
- Focal class: >50% normal glomeruli
- Crescentic class: >50% glomeruli with cellular crescents
- Mixed class: <50% normal, <50% crescentic, <50% globally sclerotic glomeruli.

To assess the MCCS [14] the following parameters were used:

- Global and segmental glomerulosclerosis was scored from 0 to 3 (0 in <10%; 1 when between 10% and 25%; 2 between 26% and 50%; 3 in >50% of sclerotic glomeruli)
- Tubular atrophy and interstitial fibrosis were scored both from 0 to 3 (0 when <10%; 1 when 10%–25%; 2 when 26%–50%; 3 when >50% of the renal cortex)
- Arteriosclerosis was scored 0 when the intima was thickened less than the media; 1 when vice versa

The total chronicity score was defined as “minimal” when it was 0, “mild” when between 2 and 4, “moderate” between 5 and 7, and “severe” when >8.

The RRS [15] was assessed by grading the following features:

- Percentage of normal glomeruli: 0 points when >25%; 4 points between 10% and 25%; 6 points <10%
- Percentage of renal cortex with tubular atrophy and interstitial fibrosis (0 points ≤25%; 2 points >25% of the cortex)
- eGFR (CKD-EPI method [17]) at the time of biopsy: 0 points for eGFR > 15 mL/min; 3 points for eGFR ≤15 mL/min

The total score, resulting from summing the points attributed to each criterion, ranged from 0 to 11. Patients were divided into three risk groups: low (0 points), medium (2–7 points) and high (8–11 points).

### Definition and endpoints of the study

- MPO- and PR3-ANCA were tested by enzyme-linked immunosorbent assay.
- We classified patients as MPA, GPA, EGPA or renal limited forms using the European Medicines Agency algorithm [18].
- Urinary sediment was considered active when >5 red blood cells/high power field were present in absence of infection or other causes.
- Arterial hypertension was defined as a person's systolic blood pressure ≥140 mmHg and/or diastolic blood pressure ≥90 mmHg following repeated examinations [19].
- The diagnosis of ANCA-GN was set following the last validated classification criteria [20–22].
- BVAS was calculated using Birmingham Vasculitis Activity Score [16].
- eGFR was calculated with the CKD-EPI method [17].
- Chronic kidney disease (CKD) stage was defined following the KDIGO definition: eGFR <60 mL/min/1.73/m^2^ or markers of kidney damage for >3 months [23, 24].
- The first endpoint was ESKD defined as CKD stage 5 and/or the need for chronic kidney replacement therapy.
- The second endpoint was ESKD or death.

We have also performed a separate analysis in the subgroup of patients who required chronic kidney replacement therapy and in patients who died.

This retrospective analysis was conducted in respect of the Helsinki Declaration and was approved by the Ethics Committee of the IRCCS Fondazione San Gerardo dei Tintori, Monza, Italy (protocol number 1922).

### Statistical analysis

We described categorical variables of demographic and clinical data as numbers (percentages, %) and we used mean value ± standard deviation (SD) or median and interquartile range (IQR) for continuous variables depending on their distribution. We applied T-test to continuous variables with normal distribution and Mann–Whitney U-test to continuous variables without a normal distribution. For categorical variables Fisher's test was applied. All the clinical/histological variables reported in Tables 1a and 1b were tested, using the Cox proportional hazard model, to find the predictors of ESKD. Both uni- and multivariable analyses have been performed. Survival curves were drawn using the Kaplan–Meier estimate and compared using the log-rank test.

**Table 1a:** Clinical characteristics at diagnosis of AAV-GN and outcome of all patients, and of whose who developed or did not develop ESKD.

Clinical characteristics	All (*n* = 152)	ESKD (*n* = 59)	No ESKD (*n* = 93)	*P*-value
Male, *n* (%)	84 (55.3)	33 (55.0)	51 (55.4)	.89
Age, years	63.8 (51.3–70.8)	66 (53.7–74.8)	62.0 (47.9–70.0)	**.03**
Serum creatinine, mg/dL	3.9 (2.45–7.0)	6.0 (4.0–8.2)	3.1 (2.0–4.7)	**.001**
Proteinuria g/day, mean (SD)	1.53 (1.66)	2.05 (1.96)	1.24 (1.29)	**.005**
Hemoglobin, g/dL	9.00 (8.1–10.4)	8.7 (8.2–9.8)	9.3 (8.1–10.7)	**.048**
eGFR, mL/min/1.73 m^2^	13.0 (7.0–26.0)	7.7 (6.0–13.6)	18.0 (11.2–32.5)	**.0001**
eGFR <15 mL/min/1.73 m^2^, *n* (%)	86 (56.5)	46 (77.9)	40 (43.0)	**.000023**
GPA, *n* (%)	55 (36.2)	20 (33.8)	35 (37.6)	.64
MPA, *n* (%)	66 (43.4)	25 (42.3)	41 (44.0)	.72
Renal-limited vasculitis, *n* (%)	30 (19.7)	14 (23.7)	16 (17.2)	.32
MPO-ANCA, *n* (%)^a^	77 (50.6)	28 (47.4)	49 (52.6)	.39
PR3-ANCA, *n* (%)	49 (32.2)	20 (33.8)	29 (31.1)	.72
BVAS	15.0 (13.0–19.0)	15 (12.5–19.0)	15.0 (13.0–19.0)	.95
MPO-ANCA and PR3-ANCA, *n* (%)	3 (1.9)	1 (1.7)	2 (2.1)	.84
Cutaneous involvement, *n* (%)	14 (9.2)	5 (8.4)	9 (9.6)	.06
Joint involvement, *n* (%)	36 (23.6)	15 (25.4)	21 (22.5)	.48
Ear nose and throat involvement, *n* (%)	25 (16.4)	8 (26.6)	17 (18.2)	.44
Pulmonary involvement, *n* (%)	28 (18.42)	8 (13.5)	20 (21.5	.21
Gastrointestinal involvement, *n* (%)	1 (0.6)	1 (1.6)	0	
Hypertension, *n* (%)	80 (52.6)	39 (66.1)	41 (44.5)	**.008**
Follow-up, months	46.9 (12.8–119.0)	11.3 (3.3–51.9)	74.1 (33.4–140.9)	**<.00001**
Induction therapy				
MP pulses, *n* (%)	136 (89.4)	53 (89.8)	83 (89.2)	.909
Immunosuppressants^b^, *n* (%)	102 (67.1)	37 (62.7)	65 (69.8)	.358
Plasma exchange^c^, *n* (%)	19 (12.4)	8 (13.5)	11 (11.8)	.753
Maintenance therapy				
Prednisone^d^, *n* (%)	41 (27.0)	13 (22.0)	28 (30.1)	.27
Immunosuppressant^e^, *n* (%)	69 (45.4)	16 (27.1)	53 (57.0)	**.0003**
Deaths, *n* (%)	39 (25.4)	20 (33.9)	19 (20.4)	.06

If not otherwise specified, data are reported as median and IQR.

^a^Data non available for 11 patients.

^b^Eighty-three patients received cyclophosphamide (32 developed ESKD); 18 received rituximab (5 developed ESKD); 1 received methotrexate.

^c^Fifteen patients received plasma exchange after an immunosuppressant agent.

^d^Patients who have received only prednisone as maintenance therapy for at least 6 months.

^e^Patients treated with steroid for at least 6 months and immunosuppressant. Forty-four patients received azathioprine at the beginning of maintenance therapy; 11 patients received mycophenolate mofetil; 12 patients received rituximab; 1 received methotrexate; 1 received cyclophosphamide.

*P* < .05 is considered significant (Pearson chi-square test for categorical variables, analysis of variance for continuous variables) and is shown in bold.

MP, methylprednisolone.

**Table 1b:** Histological characteristics at diagnosis of AAV-GN of all patients and of whose who developed or not developed ESKD.

Histological characteristics	All (*n* = 152)	ESKD (*n* = 59)	no ESKD (*n* = 93)	*P*-value
Total glomeruli *n*	17 (12–25.3)	17 (12–25.5)	17 (12–25)	0.48
Percentage of normal glomeruli	21.4 (7.4–40)	9 (0–23.5)	29 (14–50)	**.000056**
Percentage of glomerular hyalinosis	16.1 (7–33)	19.4 (8–41.2)	14 (5.7–29)	**.031**
Percentage of glomeruli with extracapillary proliferation	27.6 (14–58)	29.6 (20.5–66.3)	26 (12.5–50)	.82
Percentage of glomeruli with sclerotic extracapillary proliferation, mean (SD)	9.2 (13.9)	10.4 (14.6)	8.3 (13.5)	.35
Specimen with endoarteritis, *n* (%)	7 (4.6)	4 (6.8)	3 (3.2)	.31
Specimen granulomatous interstitial nephritis, *n* (%)	47 (30.9)	20 (33.9)	27 (29)	.54
Specimen with interstitial nephritis, *n* (%)	113 (74.3)	48 (81.4)	65 (69.9)	.1
Specimen with tubulitis, *n* (%)	31 (20.4)	14 (23.7)	17 (18.3)	.41
Grade of interstitial fibrosis and tubular atrophy, *n* (%)				**.039**
Absent	33 (21.7)	11 (18.6)	22 (23.7)	
Mild	55 (36.2)	15 (25.4)	40 (43)	
Moderate	32 (21.1)	18 (30.5)	14 (15.1)	
Severe	30 (19.7)	14 (23.7)	16 (17.2)	

If not otherwise specified, data are reported as median and IQR.

The predictive accuracy of the three classification tools was assessed by receiver opertating characteristic (ROC) curves for overall adverse outcomes (ESKD and death) and renal survival alone (ESKD). We calculated 95% confidence intervals (CIs) of area under the curve (AUC) of ROC, and of their difference, along with the Wald tests using Stata programs roctab and roccomp (Stata release 18, 2023, StataCorp, College Station, TX, USA) with default standard errors which are based an algorithm suggested by Delong and Clarke-Pearson [25].

## RESULTS

### Characteristics at baseline and at outcome of the whole cohort

Clinical and biochemical characteristics at ANCA-GN presentation of all patients and of those who developed or did not develop ESKD are reported in Table 1a. Of the 152 patients selected for the study, 84 (55.3%) were male, the median age was 63.8 (IQR 51.3–70.8) years. Median eGFR at diagnosis was 13.0 (7.0–26.0) mL/min/1.73 m^2^. Histological characteristics at diagnosis are summarized in Table 1b.

After a median follow-up time of 46.9 months (IQR 12.8–119), 59 patients (38.8%) developed ESKD and 39 patients (25.4%) died, among which 20 patients died after having reached ESKD.

The ESKD pure survival rate was 79% at 1 year, 65% at 5 years and 59.8% at 10 years. Patients’ survival was 93.8% at 1 year, 79.8% at 5 years and 65% at 10 years. The ESKD/death free survival rate (second endpoint) was 78% at 1 year, 58.3% at 5 years and 47.4% at 10 years.

### Predictors of ESKD at multivariate analysis

The results at univariate analysis are reported in Supplementary data, Table S1. At multivariate analysis (Table 2), when we considered the baseline clinical and histological features only, arterial hypertension (OR = 2.75, 95% CI 1.50–5.06; *P* = .0011), serum creatinine (OR = 1.17 for any mg increase in serum creatinine, 95% CI 1.09–1.25; *P* < .0001) and the percentage of normal glomeruli (OR = 0.97, 95% CI 0.96–0.99; *P* = .009) were the independent predictors of ESKD. When the three histological scores were added to the clinical/histological features, we found that in addition to arterial hypertension (OR = 2.73, 95% CI 1.48–5.06; *P* = .00149) and serum creatinine (OR = 1.22, 95% CI 1.12–1.32; *P* < .0001), the RRS (OR = 2.32, 95% CI 1.25–4.32; *P* = 0.0081) and the MCCS (OR = 1.06, 95% CI 1.06–3.96; *P* = .0392), but not Berden score (OR = 1.1764, 95% CI 0.6218–2.2256; *P* = .6193), were independent predictors of ESKD.

**Table 2:** Predictors, at multivariate analysis, of ESKDs among clinical histological features at diagnosis of ANCA-GN.

	Multivariate analysis 1			Multivariate analysis 2		
	OR	CI	*P*	OR	CI	*P*
Age, years						
Arterial hypertension	2.7573	1.5025–5.0601	.0011	2.7214	1.4585–5.0780	.0017
Serum creatinine, mg/dL	1.1694	1.0933–1.2509	.0000	1.2059	1.1080–1.3125	.0000
% Normal glomeruli	0.9774	0.9609–0.9942	.0090			
Berden^a^				1.1764	0.6218–2.2256	.6193
RRS^b^				2.2146	1.1558–4.2434	.0171
MCCS^c^				2.0320	1.0444–3.9533	.0378

^a^BERDEN, Berden classification, crescentic + sclerotic vs focal + mixed.

^b^RRS, Renal Risk Score, high vs low + medium.

^c^MCCS, Mayo Clinic Chronicity Score, severe + moderate vs minimal + mild.

### Characteristics of the three different classification scores

#### Berden histological classification

According to Berden histopathological classification 29 patients (19.1%) were classified as focal, 43 (28.3%) as crescentic, 58 (38.1%) as mixed and 22 (14.45%) as sclerotic. Sclerotic and crescentic groups had the worst clinical presentation in terms of renal dysfunction than mixed and focal classes. Crescentic form has the highest BVAS. MPA was more frequent in sclerotic and in mixed classes while GPA was more frequent in focal and in crescentic classes.

**Figure 1:**
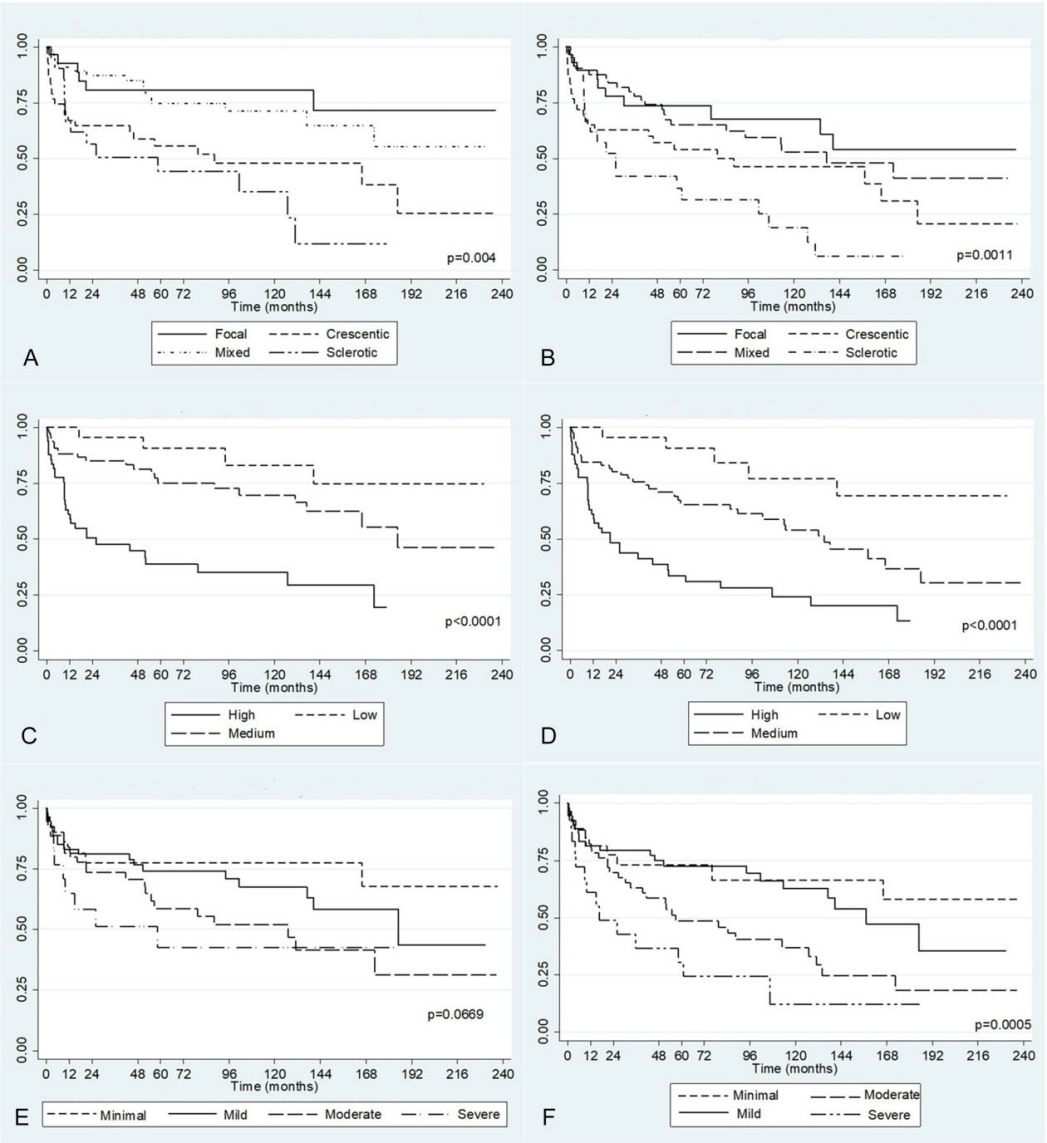
Kaplan–Meier survival curves for primary and secondary endpoint of Berden histological classification (**A, B**), RRS (**C, D**) and MCCS (**E, F**).

Figure 1A shows the Kaplan–Meier curve for the first endpoint (ESKD). The kidney survival worsens progressively from focal, to mixed, to crescentic and to sclerotic. Crescentic and sclerotic groups had the worst outcome, with ESKD free survival at 10 years of 47.8 and 33.5%, respectively, in comparison with 80% for focal and 70% for mixed classes (*P* = .004). For the second endpoint (ESKD or death) (Fig. 1B) we found the worst survival for the sclerotic group; it was 17.9% at 10 years, in comparison with 46.3% in crescentic, 52.0% in mixed and 67.5% in focal classes (*P* = .0011) (Supplementary data, Table S2a).

#### Renal Risk Score

Twenty-four patients (15.7%) were classified in the low-risk group, 78 (51.3%) in the medium-risk group and 50 (32.9%) in the high-risk group (Supplementary data, Table S2b). Median RRS at diagnosis was 6 (IQR 3–9). Applying this classification, we observed more severe renal dysfunction at ANCA-GN diagnosis in the high-risk group in comparison with the low-risk group (*P* < .0001).

Kaplan–Meier curves for first and second endpoint (Fig. 1C and D) demonstrate statistically significant differences among the three groups with the best outcome for low- and the worst for high-risk groups. For the low-risk group, the pure kidney survival rate at 10 years was 83.1%, it was 68.9% for the medium-risk and 34.4% for the high-risk group (*P* < .0001). For the second endpoint survival at 10 years was 77.1% for the low group, 53.3% for the medium and 23.4% for the high group (*P* = .0001).

#### Mayo Clinic Chronicity Score

Biopsy specimens were classified according to the MCCS into four groups (Supplementary data, Table S2c): minimal (27 patients; 17.7%), mild (54 patients; 35.6%), moderate (52 patients; 34.2%), severe (19 patients; 12.5%).

Survival curves for the first endpoint (Fig. 1E) do not show statistically significant differences among the four groups (*P* = .066). Instead, the survival free from ESKD at 10 years of minimal + mild groups (70%) was significantly better than that of moderate + severe groups (47.8%, *P* = .017). Otherwise, the survival curves for the second endpoint (ESKD or death) (Fig. 1F) were significantly different among the four groups. The survival rate at 10 years for minimal, mild, moderate and severe classes was 66.3%, 62.7%, 36.1% and 11.6%, respectively (*P* = .0005).

### Separate analysis in patients who required chronic replacement therapy and/or died during follow-up

**Figure 2:**
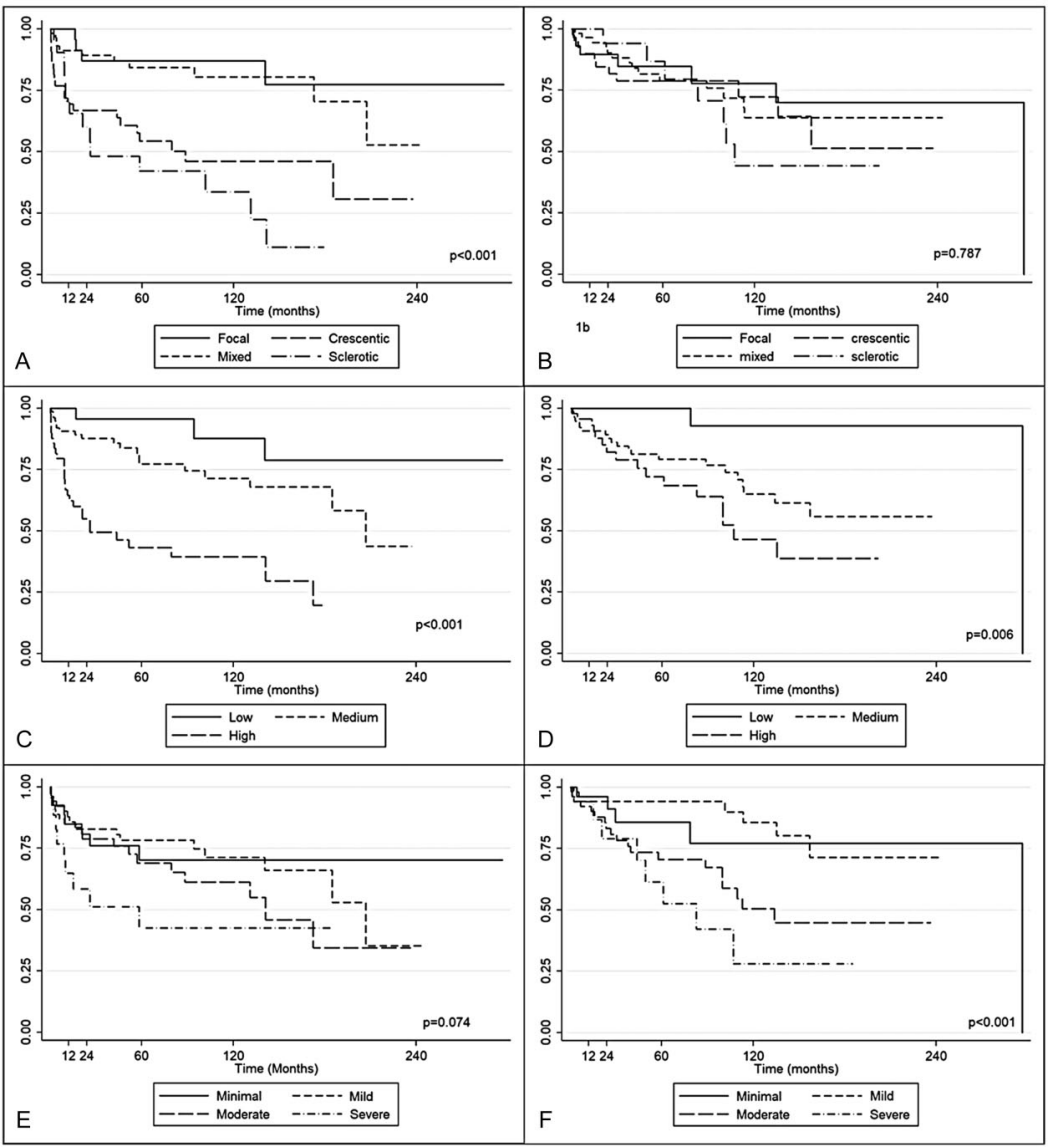
Kaplan–Meier survival curves for patients who required hemodialysis and for patients who died at the end of the follow-up, respectively, for Berden histological classification (**A, B**), RRS (**C, D**) and MCCS (**E, F**).

Since a few studies have analyzed the predictive value of the three scoring systems in predicting patient survival [8, 26] we performed a sub-analysis dividing patients who reached ESKD requiring kidney replacement therapy from those who died from any causes. Only nine patients reached stage 5 CKD without starting dialysis at last observation and therefore this subgroup was excluded from this sub-analysis. In Fig. 2 the Kaplan–Meier curves for the three histological tools, in patients requiring kidney replacement therapy (Fig. 2A) and in those who died (Fig. 2B), are reported. Berden classification and RRS groups were confirmed to be associated with the development of ESKD requiring kidney replacement therapy (*P* < .0001 with both tools), while MCCS was not (*P* = .07). For MCCS, we noticed that renal outcome for minimal + mild groups was not significantly better than that of moderate + severe groups (*P* = .055).

Considering mortality as the endpoint, Berden classification was not associated with death (*P* = .79), while RRS and MCCS showed a better discrimination power (*P* = .006 and .0008, respectively) for this outcome.

### Comparison, among the three scores, at ROC curves for the association with primary and secondary endpoints

For the first endpoint, at ROC analysis, the area under curve of RRS [AUC 0.696, confidence limits (CL) 0.618–0.775] and that of MCCS (AUC 0.605, CL 0.517–0.692) had low accuracy, and that of Berden classification (AUC 0.581, CL 0.490–0.672) was not significant (Table 3).

**Table 3:** Comparison of the AUCs at ROC analysis of histologic classification, RRS and MCCS for the different endpoints of the study.

	AUC at ROC curves analysis		
	ESKD	ESKD plus death	ESKD at 10 years
Berden histopathologic classification	0.581	0.599	0.573
RRS	0.696	0.679	0.710
MCCS	0.605	0.662	0.617

**Figure 3:**
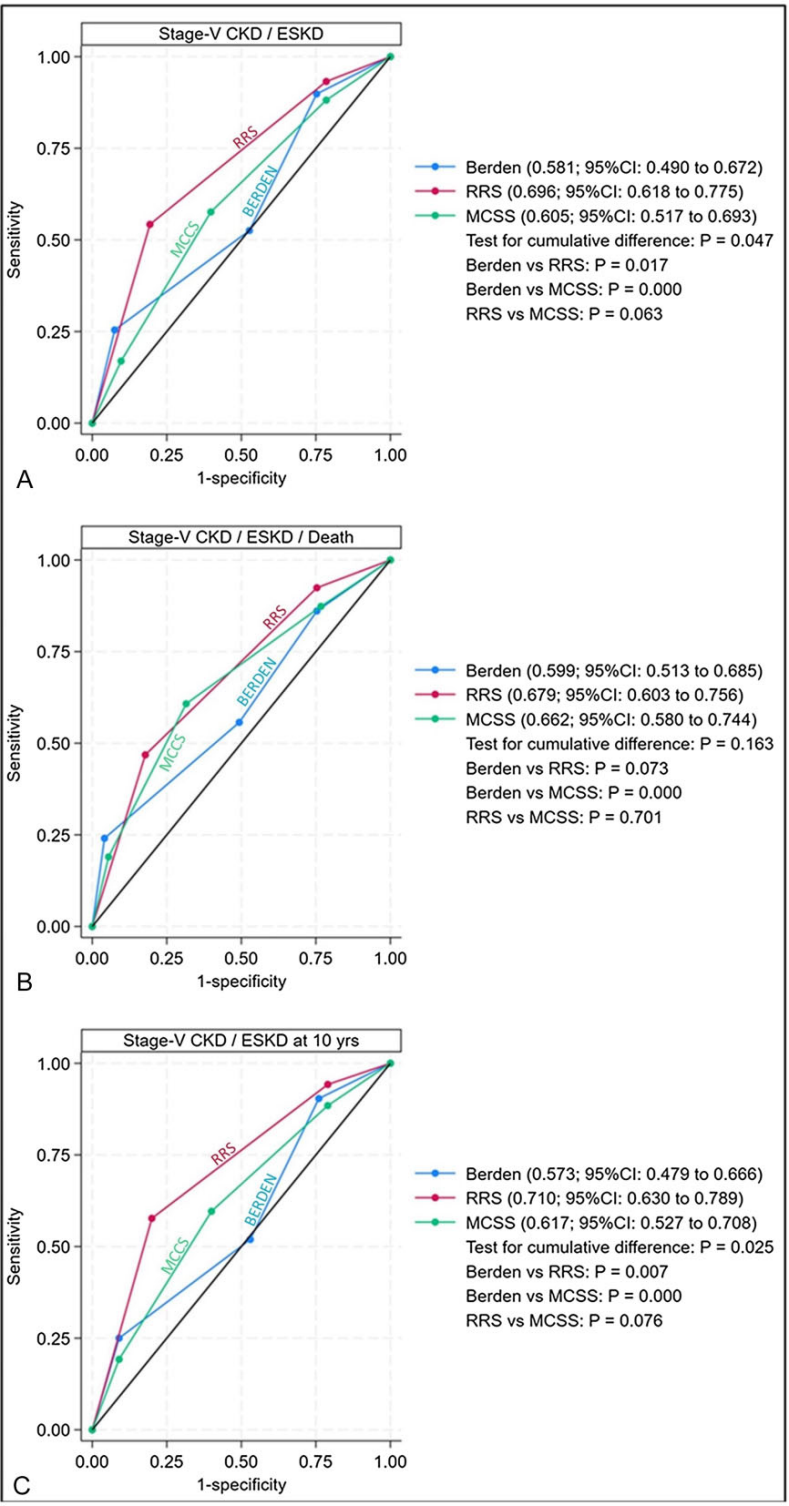
ROC curves for Berden histopathologic classification, RRS and MCCS, for primary outcome (**A**) and secondary outcome (**B**) at the end of follow-up, and for primary outcome at 10 years (**C**). *P*-values are shown beside figures.

Notwithstanding the potential limited value of this analysis, among the three scores, the test for cumulative difference for ESKD was significant (*P* = .047). The discriminative power of RRS was not significantly different from that of MCCS, being 0.091 (95% CI –0.005 to –0.020; *P* = .063), although a trend in favor of RRS was present; the worst results were achieved by Berden score [Berden vs RRS: –0.115 (95% CI –0.210 to –0.020; *P* = .017); and vs MCCS: –0.024 (95% CI –0.115 to 0.067; *P* = .000)] (Fig. 3A).

For the second endpoint, the AUCs of RRS (AUC 0.679, CL 0.603–0.756) and of MCCS (AUC 0.662, CL 0.579–0.744) had low accuracy and that of Berden score (AUC 0.599, CL 0.513–0.684) was not significant (Table 3). No cumulative difference was present among the three scores (*P* = .163) (Fig. 3B).

## DISCUSSION

Renal involvement and its clinical/histological severity are the most important predictors of bad patient and kidney prognosis in AAV [6, 27, 28]. In our cohort of ANCA-GN patients we found that the pure kidney survival rate was 65% at 5 years and 59.8% at 10 years, in line with what has been described by other authors [10, 29]. After a median follow-up time of 46.9 months, 25% of our patients had died. These results underline that despite improvement in the therapeutic approach in recent decades of ANCA-GN [10, 29] the prognosis of the disease is still not satisfactory.

One of the aims of our study was to look for the predictors of ESKD among the clinical/histological features at time of kidney biopsy. We found that, at multivariate analysis, arterial hypertension, serum creatinine and the percentage of normal glomeruli were the independent predictors of ESKD. These results underline the importance of timely diagnosis of ANCA-GN to prevent the deterioration of kidney function and the irreversible damage of glomeruli. All these factors have been already associated with worse patient and renal outcome [6, 26, 30–33].

A histological tool that could help to better stratify the risk of a worse outcome would be of upmost importance. Until now, three different scoring systems have been developed and applied to ANCA-GN [11, 14, 15] and have been found to correlate with kidney outcome [11, 13, 15, 33–39]. To the best of our knowledge, only one study has compared the three tools, in a Chinese cohort [40], and this is the first study comparing all the three classification tools in a European case series. The main difference between Berden score and RRS and MCCS was that, in Berden classification, only active and chronic glomerular lesions were evaluated; both RRS and MCCS have instead taken into consideration glomerular and tubulointerstitial lesions. In RRS, the baseline eGFR was added to the histological features, while MCCS considers only pure chronic lesions. Several studies have demonstrated the predictive value for kidney outcome of chronic tubulointerstitial lesions [34, 41, 42].

The association between Berden's histopathological classification and kidney outcome has been well established by several authors [13, 33, 35]. Most of these studies confirmed a significant difference between focal and sclerotic class in predicting kidney survival [33, 35]; instead the prognostic role of crescentic and mixed classes was debated [43, 44]. In our study, as well as in others [13, 26, 34], patients with crescentic and sclerotic classes had the worst kidney prognosis in comparison with mixed and focal classes. No prognostic difference between crescentic and mixed classes was underlined by other authors [13, 45] and this was confirmed by a recent meta-analysis by Huang *et al*. [46].

The RRS, proposed by Brix, incorporates not only glomerular but also tubulointerstitial lesions at kidney biopsy, and added eGFR at baseline to stratify the risk of developing ESKD. We confirmed the ability of this tool to predict renal outcome. Low-, medium- and high-risk groups had significantly progressive reduced kidney survival at 10 years as demonstrated by other studies [36, 39, 42, 44, 47–49].

The MCCS was conceived to evaluate all types of glomerular diseases; it is based on the degree of glomerular, tubulointerstitial and vascular chronic lesions. In Kaplan–Meier survival analysis of MCCS classes we found a significant difference in the development of ESKD only stratifying patients in two groups: minimal/mild vs moderate/severe. Currently, few studies have tested the MCCS in ANCA-GN. Berti *et al*. [50], in a cohort of 59 patients, found better renal recovery at 1 year in minimal/mild classes in comparison with moderate/severe classes. Casal Moura and colleagues [38] showed a statistically significant difference of pure kidney survival at Kaplan–Meier analysis among the four classes at 12 months only, and for the composite outcome ESKD + death at 24 months. An *et al*. [40], in a cohort of 252 ANCA-GN patients followed for 64 months, showed that at Kaplan–Meier analysis kidney survival progressively reduced from minimal to moderate, mild and to severe classes. This Chinese study was the first and the only, to the best of our knowledge, that compared the three scoring systems for ANCA-GN and their correlation to dialysis dependence. At their ROC analysis, RRS had a moderate accurate AUC, while the AUC of MCCS had low accuracy and that of the histological score was not significant. Despite these not very good results, they conducted a sensitivity analysis of the three histological tools comparing the areas under ROC curves. RRS and MCCS had both similar discrimination power that was significantly better than that of histopathological classification. We globally obtained slightly worse results than those of this study. None of our ROC curves achieved at least moderate accuracy, and consequently the discrimination power of the sensitivity analysis of the model is very limited.

We performed a sub-analysis in patients who required kidney replacement therapy or died for any cause during the follow-up, to establish the real kidney and patient survival rate of our cohort. Based on the result of this analysis, Berden classification and RRS were confirmed to be associated to ESKD, while MCCS was not. RRS and MCCS were associated with death for any cause. This result underlines and confirms the ability of RRS to stratify these patients, both when we consider the outcome ESKD and when we consider death.

There are some differences between our and other studies. Patients with renal-limited vasculitis, which accounts for around 20% of our cases, were not included in other studies [39, 40, 47, 49]. In the cohort of An *et al*., in comparison with our cohort, more patients were MPO-ANCA positive (88% vs 50.6% respectively), younger (57.5 vs 63.8 years), and with more recent and shorter period of enrollment (from 1997 to 2018 vs 1990 to 2022).

Altogether, in the Chinese cohort, when both RRS and MCCS were added to the to the base model of dialysis dependency the discrimination power significantly improved for both histological classifications but was superior for RRS. Similarly, in our second model of multivariate analysis, when the three histological scores were added to the clinical predictive features, both RRS and MCCS emerged as independent predictors of ESKD with an OR slightly better for RRS in comparison with MCCS.

This study has some limitations. It is retrospective and the patients were diagnosed during a long period during which the therapeutic treatment of these forms has significantly changed. The great majority of patients were Caucasians, and our results cannot be extended to other ethnicities.

In conclusion, RRS and MCCS scores were independently associated with kidney survival, along with serum creatinine and arterial hypertension, at multivariate analysis. This underlines the importance of kidney extraglomerular chronic lesions. RRS showed a significant association to ESKD requiring kidney replacement therapy; the trend in favor of a better performance of this score may be attributed to addition of eGFR to the histological lesions. Both tests, RRS and MCCS, should be taken as complementary, one clinical/pathological and the other purely of chronic histological lesions.

## Supplementary Material

sfae125_Supplemental_File

## Data Availability

Original data are available from the corresponding author upon request.
