# Supplementary material for: The value of the current histological scores and classifications of ANCA glomerulonephritis in predicting long-term outcome
Source: Clin Kidney J. 2024 May 6;17(7):sfae125. doi: 10.1093/ckj/sfae125 (PMC11217817; doi:10.1093/ckj/sfae125)
Supplement: sfae125_Supplemental_File [file sfae125_supplemental_file.docx]

**Supplemental tables**

Supplemental Table 1 - Predictors, at univariate analysis, of end stage kidney diseases among clinical histological features at diagnosis of ANCA-GN

|  | Univariate analysis | | |
| --- | --- | --- | --- |
|  | OR | CI | P |
| Age years | 1.0295 | 1.0103-1.0490 | 0.0014 |
| Arterial hypertension | 3.3547 | 1.8360-6.1298 | <0.0001 |
| Serum creat. mg/dl | 1.2031 | 1.1310-1.2799 | <0.0001 |
| eGFRmL/min/1.73 m2 | 0.9436 | 0.9174-0.9705 | <0.0001 |
| Hemoglobin g/Dl | 0.8021 | 0.6811-0.9446 | 0.0076 |
| Maintenance therapy | 0.4052 | 0.2378-0.6906 | 0.0007 |
| % Normal Glomeruli | 0.9697 | 0.9549-0.9847 | <0.0001 |
| % Jaline Glomeruli | 1.0132 | 1.0014-1.0252 | 0.0366 |
| Interstitial fibrosis | 1.9593 | 1.1631-3.3004 | 0.0118 |
| %Extracapillary proliferation | 1.016 | 1.005-1.027 | 0.0056 |
| BERDEN * | 2.7495 | 1.6192-4.6686 | 0.0001 |
| RRS** | 3.7567 | 2.2172-6.3651 | 0.0001 |
| MCCS** | 1.9373 | 1.4826-3.2686 | 0.0137 |

Legend: create, creatinine; eGFR, estimated glomerular filtration rate; OR, odds-ratio; CI, confidence interval.

Supplemental Table 2 - Clinical and histological characteristics of AAV-GN patients at diagnosis and at outcome classified according to: a) Berden histopathologic classification b) the Renal Risk Score c) Mayo Clinic Chronicity Score

**2a**

| **Clinical characteristics** | **Focal (n=29)** | **Crescentic (n=43)** | **Mixed (n=58)** | **Sclerotic (n=22)** | **p_1_** | **p_2_*** |
| --- | --- | --- | --- | --- | --- | --- |
| Hypertension, n (%) | 11 (37.9) | 22 (51.1) | 32 (55.1) | 15 (68.1) | 0.18 | **0.03** |
| Serum creatinine (mg/dL) | 2.6 (1.6-5.1) | 6.2 (3.4-8.9) | 3.6 (2.2-4.7) | 5.1 (3.0-7.4) | **0.00001** | **0.01** |
| eGFR (ml/min/1.73 m2) | 28.0 (12.6-47.0) | 7.0 (5.6-16.5) | 14.9 (10.0.27.2) | 9.0 (7.0-17.7) | **0.00005** | **0.0032** |
| MPO - ANCA n (%) | 9 (31.0) | 17 (39.5) | 36 (62.1) | 15 (68.2) | **0.0063** | **0.008** |
| PR3 - ANCA n (%) | 10 (34.5) | 20 (46.5) | 14 (24.1) | 5 (22.7) | 0.081 | 0.36 |
| GPA, n (%) | 10 (34.5) | 24 (55.8) | 17 (29.3) | 4 (18.2) | **0.0093** | 0.196 |
| MPA, n (%) | 10 (34.5) | 13 (30.2) | 32 (55.2) | 11 (50.0) | 0.054 | 0.26 |
| Renal-limited vasculitis, n (%) | 9 (31.0) | 5 (11.6) | 9 (15.5) | 7 (31.8) | 0.078 | 0.952 |
| Proteinuria (g/die) | 0.7 (0.3-1.2) | 0.9 (0.4-1.5) | 1.1 (0.6-1.8) | 1.6 (0.6-2.6) | 0.07 | **0.017** |
| Percentage of normal glomeruli | 61.5 (54.0-71.0) | 12.0 (0-25.0) | 25.0 (11.1-34.9) | 2.0 (0-10.0) | **0.00001** | **0.00001** |
| Percentage of sclerotic glomeruli | 9.0 (0-15.3) | 9.0 (4.2-18.2) | 22.0 (11.2-35.0) | 56.3 (50.3-66.1) | **0.00001** | **0.00001** |
| BVAS | 14.0 (12.0-19.0) | 18.0 (15.0-21.0) | 15.0 (13.0-17.0) | 14.0 (12.0-17.0) | **0.0003** | 0.23 |
| ESKD n (%) | 6 (20.6) | 22 (51.2) | 16 (27.5) | 15 (68.1) | **0.0004** | **0.0006** |
| Death n (%) | 7 (24.1) | 11 (25.6) | 14 (24.1) | 7 (31.8) | 0.9 | 0.54 |

**2b**

| **Characteristics at baseline** | **Low (n=24)** | **Medium (n=78)** | **High (n=50)** | **p_1_** | **p_2_**** |
| --- | --- | --- | --- | --- | --- |
| Hypertension, n (%) | 11 (45.8) | 38 (48.7) | 31 (62.0) | 0.26 | 0.18 |
| Serum creatinine (mg/dL) | 1.9 (1.3-2.8) | 3.9 (2.5-6.8) | 4.8 (3.4-4.9) | **<0.00001** | **<0.0001** |
| eGFR (ml/min/1.73 m2) | 25.0 (16.0-38.0) | 13.0 (7.0-25.3) | 10.0 (6.0-15.5) | **<0.00001** | **<0.0001** |
| MPO - ANCA n (%) | 9 (37.5) | 43 (55.1) | 25 (50.0) | 0.317 | 0.312 |
| PR3 - ANCA n (%) | 9 (37.5) | 22 (28.2) | 18 (36.0) | 0.546 | 0.900 |
| GPA, n (%) | 10 (41.7) | 28 (35.9) | 17 (34.0) | 0.812 | 0.521 |
| MPA, n (%) | 9 (37.5) | 36 (46.1) | 21 (42.0) | 0.733 | 0.712 |
| Renal-limited vasculitis, n (%) | 5 (20.8) | 13 (16.7) | 12 (24.0) | 0.589 | 0.761 |
| Proteinuria (g/die) | 1.0 (0.4-1.7) | 0.7 (0.4-1.5) | 1.4 (0.7-2.3) | 0.39 | **0.0001** |
| Percentage of normal glomeruli | 54.0 (39.6-62.3) | 26.0 (16.0-40.0) | 0 (0-8.5) | **<0.00001** | **<0.00001** |
| Percentage of sclerotic glomeruli | 8.3 (4.5-16.0) | 15.7 (6.5-26.9) | 27.0 (9.3-50.0) | **<0.00001** | **0.0002** |
| BVAS | 17.0 (12.7-19.2) | 15.0 (13.0-19.0) | 15.0 (12.2.18.7) | 0.99 | 0.90 |
| ESKD n (%) | 4 (16.6) | 23 (29.5) | 32 (64.0) | **0.00002** | **0.0001** |
| Death n (%) | 2 (8.3) | 21 (25.3) | 16 (32.0) | 0.08 | **0.026** |

**2c**

| **Characteristics at baseline** | **Minimal (n=27)** | **Mild (n=54)** | **Moderate (n=52)** | **Severe (n=19)** | **p_1_** | **p_2_***** |
| --- | --- | --- | --- | --- | --- | --- |
| Hypertension, n (%) | 10 (37.0) | 26 (48.1) | 31 (59.6) | 13 (26.5) | 0.11 | **0.03** |
| Serum creatinine (mg/dL) | 3.1 (1.9-5.1) | 3.6 (2.1-8.3) | 4.6 (3.1-7.0) | 3.8 (2.6-6.6) | 0.5 | 0.69 |
| eGFR (ml/min/1.73 m2) | 18.0 (12.5-34.0) | 16.0 (6.0-32.3) | 10.5 (7.3-16.0) | 12.9 (7.1-24.5) | **0.0022** | **0.0003** |
| MPO - ANCA n (%) | 11 (40.7) | 20 (37.0) | 35 (67.3) | 11 (57.9) | **0.0105** | **0.0011** |
| PR3 - ANCA n (%) | 12 (44.4) | 20 (37.0) | 12 (23.1) | 5 (26.3) | 0.193 | **0.0405** |
| GPA, n (%) | 13 (48.1) | 25 (46.3) | 15 (28.9) | 2 (10.5) | **0.0134** | **0.0032** |
| MPA, n (%) | 9 (33.3) | 23 (42.6) | 25 (48.1) | 9 (47.3) | 0.634 | 0.298 |
| Renal-limited vasculitis, n (%) | 5 (18.5) | 5 (9.2) | 12 (23.1) | 8 (42.1) | **0.017** | **0.0.4** |
| Proteinuria (g/die) | 0.8 (0.4-1.5) | 0.9 (0.4-1.7) | 1.2 (0.6-2.2) | 1.00 (0.5-1.9) | 0.76 | **0.79** |
| Percentage of normal glomeruli | 29.0 (11.3-60.1) | 25.0 (10.4-40.0) | 16.0 (7.4-35.0) | 9 (0-21.7) | **0.003** | **0.003** |
| Percentage of sclerotic glomeruli | 5.0 (0-10.3) | 11.1 (6.0-20.0) | 25.5 (14.0-38.0) | 44.0 (32.1-56.4) | **<0.0001** | **<0.0001** |
| BVAS | 16.0 (13.0-19.0) | 17.0 (14.0-21.0) | 15.0 (12.0-17.0) | 13.0 (12.0-15.0) | **0.01** | **0.002** |
| ESKD n (%) | 10 (37.0) | 21 (38.8) | 33 (63.5) | 15 (78.9) | **0.002** | **0.0003** |
| Death n (%) | 5 (18.5) | 7 (13.0) | 19 (36.5) | 8 (42.1) | **0.01** | **0.001** |

If not otherwise specified, data are reported as median and interquartile range.
Legend: AAV-GN, ANCA associated vasculitis with glomerulonephritis; n, number; eGFR, glomerular filtration rate; BVAS, Birmingham Vasculitis Activity Score; ESKD, end-stage kidney disease; p_1,_ p-value among all classes; p_2_*, p-value among focal and sclerotic classes. p_2_**, p-value among low group and high group. p_2_***, p-value calculated by comparison of minimal plus mild classes (81 patients) versus moderate plus severe classes (71 patients).
P < 0.05 is considered significant (Pearson chi-square test for categorical variables, ANOVA for continuous variables) and is shown in bold.
